# *In Vivo* Water Dynamics in *Shewanella oneidensis* Bacteria at High Pressure

**DOI:** 10.1038/s41598-019-44704-3

**Published:** 2019-06-18

**Authors:** Fabrizia Foglia, Rachael Hazael, Filip Meersman, Martin C. Wilding, Victoria García Sakai, Sarah Rogers, Livia E. Bove, Michael Marek Koza, Martine Moulin, Michael Haertlein, V. Trevor Forsyth, Paul F. McMillan

**Affiliations:** 1 Chemistry Department, Christopher Ingold Laboratories, University College London, 20 Gordon Street, London, WC1H 0AJ UK; 2 Survivability and Advanced Materials group, Centre for Defence Engineering, Cranfield University at the Defence Academy of the UK, Shrivenham, SN6 8LA UK; 3 Biomolecular & Analytical Mass Spectrometry, Department of Chemistry, University of Antwerp, Groenenborgerlaan 171, B-2020 Antwerp, Belgium; 4 Materials Engineering, Sheffield Hallam University, Howard Street, Sheffield, S1 1WB UK; 5 ISIS Neutron and Muon Source, Rutherford Appleton Laboratory, Chilton, OX11 0QX UK; 6 Dipartimento di Fisica, Università di Roma “La Sapienza”, 00185 Roma, Italy; 7 Institut de Minéralogie, de Physique des Matériaux et de Cosmochimie, CNRS UMR 7590, Université Pierre et Marie Curie, F-75252 Paris, France; 8 Institut Laue Langevin, 6 Rue Jules Horowitz, BP 156, 38042 Grenoble, Cedex France; 9 Life Sciences Group, Carl-Ivar Brändén Building, Institut Laue-Langevin, 71 avenue des Martyrs, 38042 Grenoble, cedex 9 France; 10 Faculty of Natural Sciences/ISTM, Keele University, Staffordshire, ST5 5BG UK

**Keywords:** Biopolymers in vivo, Biological physics

## Abstract

Following observations of survival of microbes and other life forms in deep subsurface environments it is necessary to understand their biological functioning under high pressure conditions. Key aspects of biochemical reactions and transport processes within cells are determined by the intracellular water dynamics. We studied water diffusion and rotational relaxation in live *Shewanella oneidensis* bacteria at pressures up to 500 MPa using quasi-elastic neutron scattering (QENS). The intracellular diffusion exhibits a significantly greater slowdown (by −10–30%) and an increase in rotational relaxation times (+10–40%) compared with water dynamics in the aqueous solutions used to resuspend the bacterial samples. Those results indicate both a pressure-induced viscosity increase and slowdown in ionic/macromolecular transport properties within the cells affecting the rates of metabolic and other biological processes. Our new data support emerging models for intracellular organisation with nanoscale water channels threading between macromolecular regions within a dynamically organized structure rather than a homogenous gel-like cytoplasm.

## Introduction

Understanding the behavior of water inside cells is a long-standing problem in molecular biochemistry and cell biology^1–8^. The influence of high hydrostatic pressure on cellular structure and dynamics is important for areas ranging from biology in deep planetary environments to food preservation and biotechnology^9–24^. Investigating pressure effects on intracellular water dynamics forms a key aspect of this research and it leads to new insights into biological functioning of organisms at ambient and under hyperbaric conditions^25^. Water management inside prokaryotic organisms is still not completely understood. It has long been assumed that the internal structure of bacterial cells consisted of unencapsulated nucleic acid material suspended in a homogeneous gel-like mixture of proteins and other macromolecules along with aqueous electrolyte solutions forming the cytosol. Within the context of that model, main questions related to water mobility evolved around the “crowded” nature of the macromolecular environment, and how dissolved ions, metabolites and macromolecular species would affect the relaxation dynamics and transport properties. Recent discussions now suggest a more structured internal environment for the prokaryotic cell^8,26–29^. The new models envisage a dynamically changing but cooperatively well organized “superclustered” arrangement of proteins and macromolecular complexes, separated by channels containing the aqueous electrolyte solution^28^. Our new results determining intracellular water transport dynamics at high pressure support this view.

Nuclear magnetic resonance (NMR) and quasi-elastic neutron scattering (QENS) studies at ambient pressure have demonstrated diffusional dynamics for intracellular water that are indistinguishable from bulk water or aqueous solutions over length scales extending from localized environments up to cellular dimensions^30,31^. ^2^H NMR spin relaxation data also reveal ~15% water molecules that are strongly bound to internal surfaces, or buried within protein complexes^30^. Our QENS results at high pressure probe the dynamics of the main fraction of the mobile aqueous species that are responsible for transport and reaction processes within the bacterial cells, over real space correlation lengths extending between ~3 to 60 Å.

QENS provides information on diffusive and rotational relaxation rates in water and aqueous solutions over picosecond (ps) to nanosecond (ns) timescales, and techniques have now been developed for *in situ* QENS studies at high pressures extending into the GigaPascal range^32,33^. In a previous study we applied high-P QENS combined with H/D isotopic substitution of live organisms to investigate water dynamics in wild type (*WT*) *Shewanella oneidensis* at ambient and P = 200 MPa^25^. These results provided a first indication of diffusional slowdown occurring for intracellular water compared with aqueous hydrogenated or perdeuterated (*Hb*, *Db*) buffer solutions. However, it is known that H_2_O self-diffusion in aqueous media is also affected by pressure (P) as well as temperature (T) variables in this pressure range, and also depends on the presence of dissolved ions^34,35^. Here we extended the pressure range of our investigations of intracellular water dynamics up to 500 MPa to fully demonstrate the occurrence of the effect, obtaining QENS data at three separate neutron facilities and QENS instruments with different energy- and momentum-exchange and resolution characteristics. The consistency of the results clearly establishes the pressure induced changes in translational diffusion and rotational relaxation of intracellular water molecules, relative to bulk aqueous solutions used as the suspension media for the microbial samples. As part of our study, we also obtained data for pressure-resistant (*PR*) survivor populations that had been previously exposed to pressures of 500 (in a single compression step) and 750 MPa (*via* sequential intermediate treatments at 250 and 500 MPa, followed by resuscitation and re-compression) and compared the results with those for wild-type (*WT*) *S. oneidensis* samples^36,37^ (Table 1).

**Table 1:** Diffusion parameters obtained from fits by applying the jump model^38^ to *Γ*_*Τ*_(*Q*^*2*^) data obtained at all three instruments for *Hb*, *Db*, WT and PR bacterial samples at 0.1, 200 and 500 MPa.

Isotopic composition	Sample	Pressure	Energy resolution	D_t_ x 10^−5^ (cm^2^/s)	τ_0_ (ps)	τ_R_ (ps)
Normal H PBS buffer	*Hb*	0.1 MPa	85 μeV	2.65 ± 0.22	1.0 ± 0.10	2.06 ± 0.08
			60 μeV	2.70 ± 0.20	1.0 ± 0.10	2.05 ± 0.05
			17.5 μeV	2.65 ± 0.21	1.0 ± 0.09	2.00 ± 0.07
		200 MPa	60 μeV	2.40 ± 0.19	1.22 ± 0.13	2.35 ± 0.07
			17.5 μeV	2.50 ± 0.20	1.12 ± 0.09	2.20 ± 0.06
		500 MPa	85 μeV	2.30 ± 0.12	1.34 ± 0.09	2.75 ± 0.07
			17.5 μeV	2.30 ± 0.14	1.42 ± 0.11	2.64 ± 0.05
Perdeuterated PBS buffer	*Db*	0.1 MPa	85 μeV	2.10 ± 0.10	1.00 ± 0.09	2.12 ± 0.07
			60 μeV	2.00 ± 0.11	1.02 ± 0.09	2.10 ± 0.07
			17.5 μeV	2.00 ± 0.12	1.00 ± 0.08	2.09 ± 0.08
		200 MPa	85 μeV	1.70 ± 0.16	1.10 ± 0.13	2.53 ± 0.07
			60 μeV	1.80 ± 0.20	1.15 ± 0.16	2.44 ± 0.08
		500 MPa	85 μeV	1.50 ± 0.14	1.54 ± 0.11	2.99 ± 0.09
			17.5 μeV	1.40 ± 0.18	1.60 ± 0.09	2.93 ± 0.07
(*Hc/Db)* − *Db Im*: “Intracellular medium”	*WT*	0.1 MPa	60 μeV	2.57 ± 0.40	2.04 ± 0.10	2.05 ± 0.09
			17.5 μeV	2.44 ± 0.30	2.02 ± 0.90	2.06 ± 0.07
		200 MPa	60 μeV	2.11 ± 0.32	2.11 ± 0.09	2.58 ± 0.10
		500 MPa	17.5 μeV	1.76 ± 0.35	2.58 ± 0.11	2.94 ± 0.09
	*500* *MPa PR*	0.1 MPa	85 μeV	2.11 ± 0.18	2.14 ± 0.11	2.31 ± 0.08
			17.5 μeV	2.22 ± 0.20	2.41 ± 0.13	2.38 ± 0.07
		200 MPa	85 μeV	1.94 ± 0.21	2.53 ± 0.09	2.53 ± 0.09
		500 MPa	85 μeV	1.48 ± 0.18	3.17 ± 0.10	2.96 ± 0.09
			17.5 μeV	1.53 ± 0.25	3.28 ± 0.11	2.94 ± 0.10
	*750* *MPa PR*	0.1 MPa	17.5 μeV	2.24 ± 0.18	2.33 ± 0.09	2.64 ± 0.08
		500 MPa	17.5 μeV	1.73 ± 0.20	2.58 ± 0.12	3.29 ± 0.10
*(Dc/Hb* − *Dc/Db)* − *Hb Imc*: “ Intracellular medium: cytoplasm only”	*WT*	0.1 MPa	60 μeV	2.43 ± 0.14	1.81 ± 0.07	—
		200 MPa	60 μeV	2.00 ± 0.12	2.00 ± 0.10	—
	*500* *MPa PR*	0.1 MPa	85 μeV	2.04 ± 0.16	1.74 ± 0.09	—
		200 MPa	85 μeV	1.67 ± 0.18	2.04 ± 0.14	—
		500 MPa	85 μeV	1.45 ± 0.14	2.57 ± 0.12	—

The derived *D*_*T*_ value and jump frequency (*τ*_*o*_) are provided for the translational diffusion obtained by analysis of the narrow Lorentzian QENS component, and the rotational relaxational time (*τ*_*R*_) was extracted from the fit of the dynamic structure factor to the broader Lorentzian function that was used to fit some of the datasets. In cases where the data did not require fitting using this additional component, *S(Q,ω)* was modelled using a single Lorentzian function and no information on the rotational relaxational times was obtained. *Hc/Db* refers to H cells resuspended in D-buffer, and the isotopic contrast combination and dataset subtraction *Hc/Db-Db* was designed to focus on water dynamics within the intracellular medium (*Im*), which includes everything contained within the cell envelope (e.g. membranes, proteins, nucleic acids, along with the cytoplasm that is expected to dominate the diffusional dynamics). The contrast *(Dc/Hb* − *Dc/Db)* − *Hb* focused on the cytoplasmic contribution (*Imc*) only.

## Results

**Figure 1:**
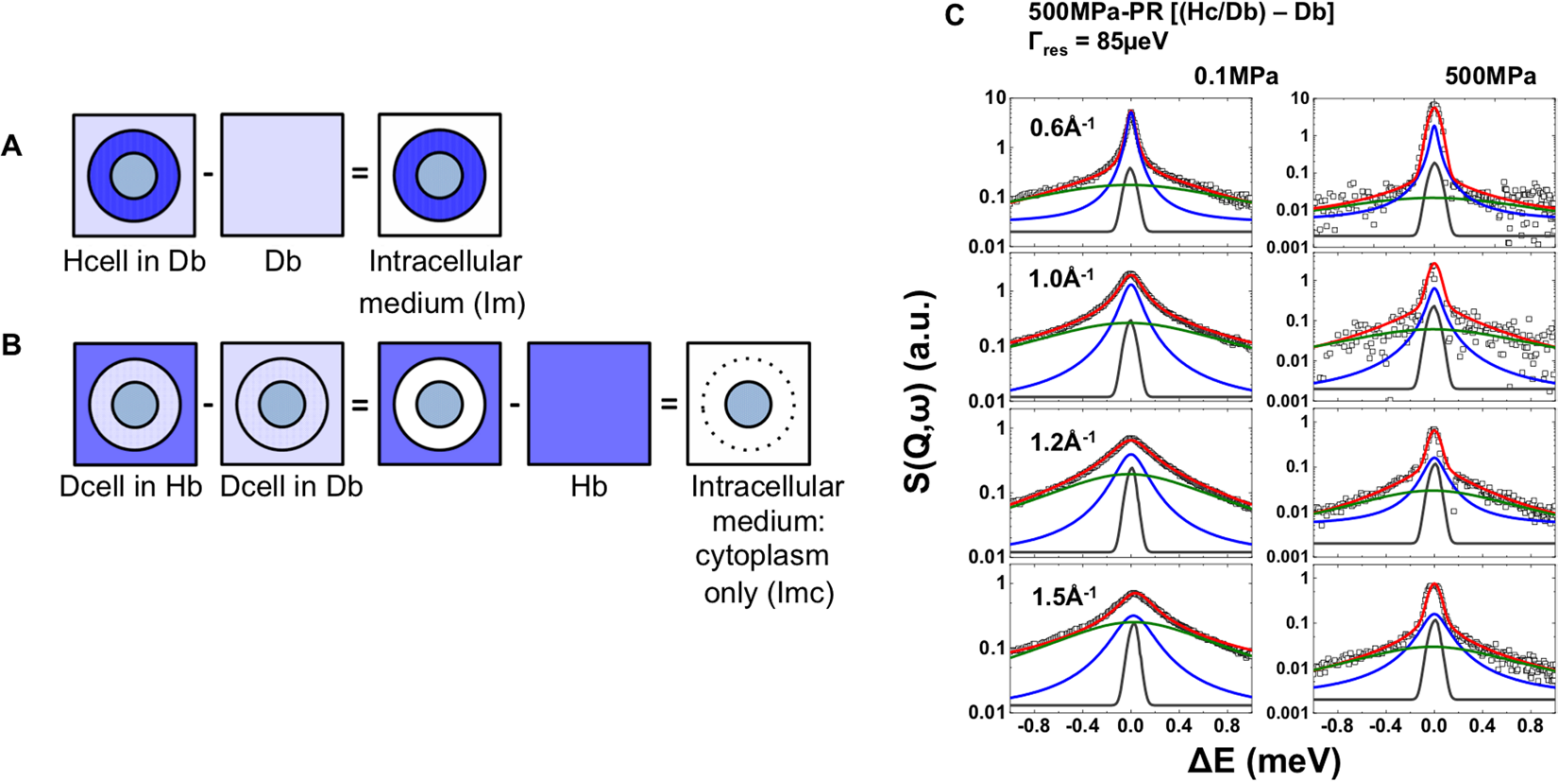
Left: Cartoon depicting isotopic QENS dataset subtractions applied to highlight intracellular water dynamics in different spatial regions. (**A**) Intracellular medium (*Im*) dynamics *via* the isotopic contrast [*Hc/Db* − *Db*] (i.e., bacterial cells minus buffer contribution). We note that this dataset subtraction provides information on the dynamics of all species contained within the cell envelope: i.e., membrane structures, proteins, DNA, RNA, etc., as well as the cytoplasm that dominates the mobile species on the QENS timescale. (**B**) Intracellular medium: cytoplasm only (*Imc*) dynamics obtained *via* the isotopic contrast [*(Dc/Hb* − *Dc/Db)* − *Hb*]. This isotopic subtraction gives information on the cytoplasm dynamics only. The two exhibit minor differences in the diffusion coefficients (Table 1). Right: (**C**) Representative datasets and analysis of the neutron dynamic scattering function *S(Q,ω)* for the intracellular medium *Im* (isotopic dataset contrast [*Hc/Db* − *Db*]). Data are shown here for a pressure-resistant *S. oneidensis* population that had been previously exposed to to 500 MPa (*500* *MPa-PR*) and subsequently cultured at ambient conditions, then examined by neutron scattering using the IN6 instrument at ILL (instrumental resolution 85 μeV; λ = 5.12 Å). Data were recorded at room temperature at pressures of 0.1 and 500 MPa for momentum transfers between 0.2–1.7 Å^−1^. The raw data and lineshape fits are shown with a logarithmic intensity scale to highlight QENS contributions. The central line (grey) due to elastic scattering is modelled by a delta function convoluted with the instrumental resolution giving rise to a Gaussian. The narrow QENS Lorentzian signal (blue line) indicates the translational diffusion component with a linewidth (*Γ*) that varies as a function of *Q*. The broader (olive-green) Lorentzian represents the faster rotational relaxation that is *Q*-independent. The global fit (red continuous curve) is overlain on the data points (black open squares). A full set of datasets obtained and their analyses is provided in Supplementary Figs 1–5.

In Fig. 1, we show results of typical QENS datasets and their analyses for WT and PR (500 MPa and 750 MPa) *S. oneidensis* bacteria at ambient (0.1 MPa), 200 MPa and 500 MPa, obtained at TOFTOF (FRM-II, Germany), IN6 (ILL, France), and IRIS (ISIS, UK)). Details of the three instruments and data analysis procedures are provided below in Methods and the full set of datasets is provided in Supplementary Information (SI). Further details of the analysis are given in our previous communication^25^. The data presented here correspond to water dynamics in the intracellular medium (*Im*) obtained from the isotopic contrast dataset combination *Hc/Db* − *Db*, using data recorded from “normal” hydrogenated cells (*Hc*) resuspended in perdeuterated buffer (*Db*), after subtracting the *Db* buffer contribution (Fig. 1). The neutron scattering (*S(Q,w)*) intensities are shown using a logarithmic scale to highlight the QENS contributions. After subtraction of the central Gaussian elastic line with its width determined by the instrumental resolution the QENS data were modelled by a narrow Lorentzian identified with translational diffusion, along with a broader second Lorentzian component from which rotational relaxation times could be extracted (Fig. 1 and Supplementary Figs 1–5).

**Figure 2:**
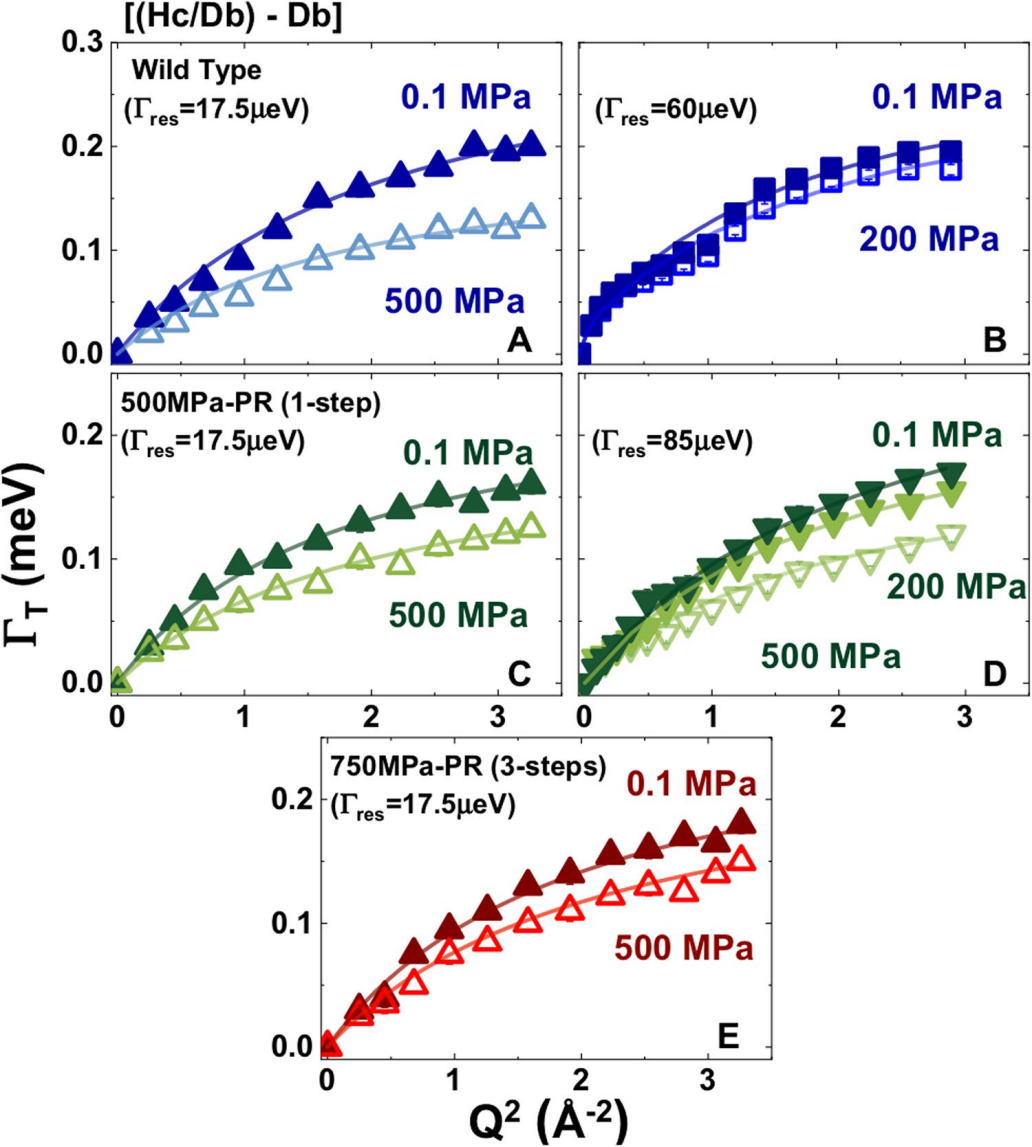
*Γ*_*T*_*(Q*^2^) data extracted from fitting QENS data of the narrow Lorentzian contribution (purely translational) from QENS data for intracellular medium (*Im* = *Hc/Db* − *Db*) for *S. oneidensis WT* (top panel), *500* *MPa-PR* (middle panel) and *750* *MPa-PR* (bottom panel) recorded at room temperature at 0.1, 200 and 500 MPa. Upward pointing triangles: data obtained at IRIS (ISIS, UK) using an instrumental resolution of 17.5 μeV: square symbols: data from TOFTOF (FRMII, Germany) at an instrumental resolution 60 μeV; downward pointing symbols: data from IN6 (ILL, France) at an instrumental resolution 85 μeV (λ = 5.12 Å). The symbols represent measured values and the solid lines show the results of fitting using a jump diffusion model with *D*_*T*_ as a fitted parameter (Table 1).

Water diffusion coefficients (*D*_*T*_) were determined by plotting the QENS half width at half maximum (HWHM, *Γ*_*T*_) for the narrow Lorentzian contribution associated with translational relaxation against the square of the reciprocal scattering vector (*Q*^2^) (Fig. 2, Table 2; Supplementary Fig. 6), and fitting the data using a jump model between sites separated by an average distance (*l*) with a mean residence time (*τ*_0_) during which the molecules undergo oscillatory motions^38^:

1 $${{\rm{\Gamma }}}_{T}=\frac{{{\rm{D}}}_{T}{Q}^{2}}{{{\rm{D}}}_{T}{Q}^{2}{\tau }_{0}+1}$$

**Table 2:** Summary of pressure effects on translational (*D*_*T*_) and rotational *(τ*_*R*_) diffusional parameters for the buffer medium (*Hb/Db*) and WT *vs* PR bacteria for data obtained at the three QENS instruments with different instrumental resolutions.

Isotopic composition	Sample	P-range (MPa)	Energy resolution	ΔD_t_ (%)	Δτ_R_ (%)
	*Hb*	0.1–200	85 μeV	~−10%	~+10%
		0.1–500	85 μeV	~−20%	~+30%
Fully H		0.1–200	60 μeV	~−10%	~+15%
		0.1–500	17.5 μeV	~−20%	~+30%
	*Db*	0.1–200	85 μeV	~−20%	~+20%
		0.1–500	85 μeV	~−30%	~+40%
Fully D		0.1–200	60 μeV	~−10%	~+10%
		0.1–500	17.5 μeV	~−30%	~+40%
	*WT*	0.1–200	60 μeV	~−20%	~+20%
		0.1–500	17.5 μeV	~−30%	~+40%
*Im*: *(Hc/Db)* − *Db*	*500* *MPa PR*	0.1–200	85 μeV	~−10%	~+10%
		0.1–500	85 μeV	~−30%	~+30%
		0.1–500	17.5 μeV	~−30%	~+20%
	*750* *MPa PR*	0.1–500	17.5 μeV	~−20%	~+30%
	*WT*	0.1–200	60 μeV	~−20%	—
*Imc*: *(Dc/Hb* − *Dc/Db)* − *Hb*	*500* *MPa PR*	0.1–200	85 μeV	~−20%	—
		0.1–500	85 μeV	~−30%	—

**Figure 3:**
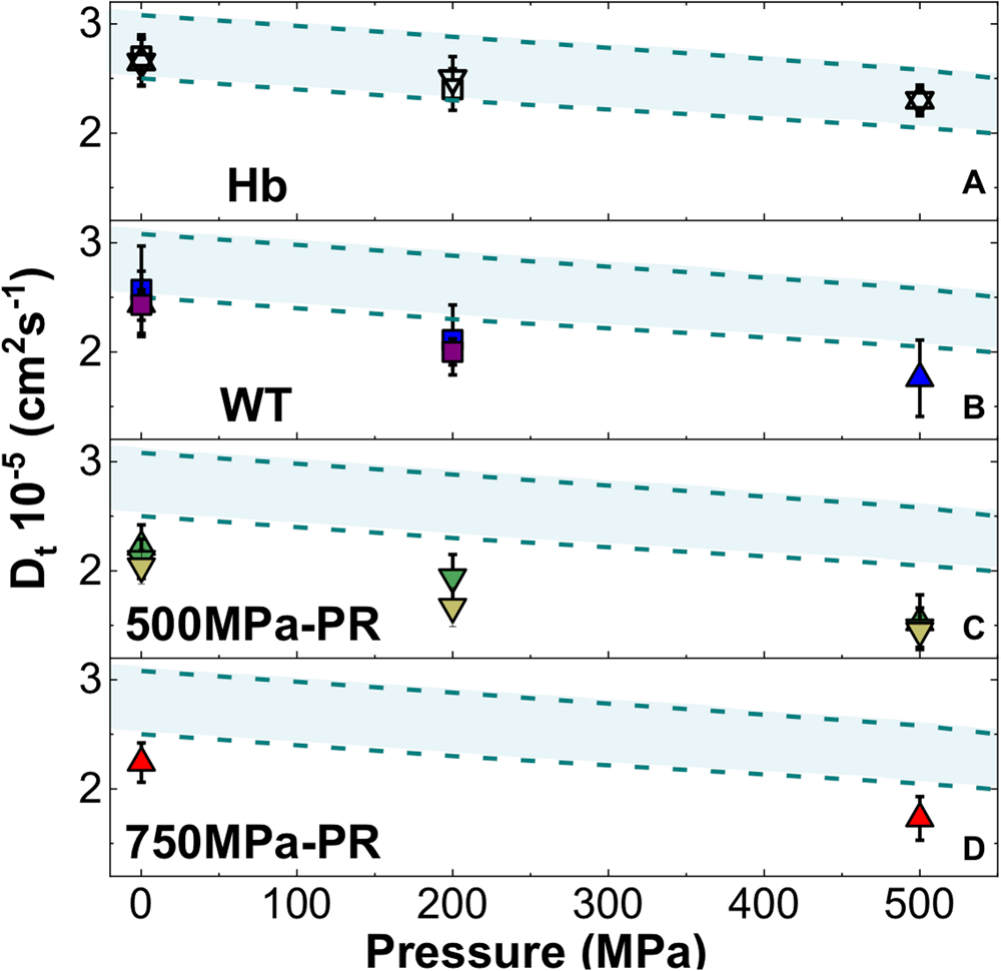
Compilation of translational self-diffusion coefficients (*D*_*T*_) extracted from QENS data obtained at the three spectrometers and neutron scattering facilities. Data from TOFTOF are shown as square symbols, IRIS as upward pointing triangles and IN6 as downward pointing triangles. Panel (A) *D*_*T*_ values for the H-buffer (Hb); panel (B) *D*_*T*_ values for intracellular [*Hc/Db* − *Db*; blue symbols] and the cytoplasm [*(Dc/Hb)* − *(Dc/Db)* − *Hb*; purple] medium for *Shewanella oneidensis WT* cells; panel (C) *D*_*T*_ values for intracellular [*Hc/Db* − *Db*; olive] and the cytoplasm [*(Dc/Hb)* − *(Dc/Db)* − *H]*; light green] medium for *S. oneidensis* 500 MPa PR cells; panel (D) *D*_*T*_ values for intracellular [*Hc/Db* − *Db*; red] medium for *S. oneidensis* 750 MPa PR cells. The dashed lines enclosing the light blue shaded area correspond to the range of literature values reported for bulk water and aqueous solutions between 298–308 K^5,31,39–42,61,68–70^.

The *Hc/Db* − *Db* isotopic contrast applied to most of our samples reveals contributions from water motions within the intracellular medium (*Im*), without distinguishing between the cytoplasm and cell envelope. The larger number of experimental runs that we could conduct at TOFTOF (FRMII) allowed us to examine a wider range of isotopic contrasts for WT samples at 1 atm and 200MPa^25^. Those data allowed construction of a further [*(Dc/Hb* − *Dc/Db)* − *Hb*] contrast that distinguishes dynamics within the cytoplasm alone (*Imc*) from those occurring within the cell envel’ (Fig. 1). That work allowed us to correlate the “dip” observed near 1 Å^−2^ in some of the *Γ*_*T*_*(Q*^2^) datasets with transmembrane water transport *via* Aquaporin channels^25^ (Fig. 2). However, the overall *D*_*T*_ values for intracellular water motions extracted from the different datasets were identical within the systematic errors associated with each measurement (Table 2). We do note, however, that the *D*_*T*_ values for the cytoplasm only (*Imc*) appear to be systematically lower (by ~5–6%) than those recorded for the full set of species contained within the cell envelope, including contributions from the membrane structures, proteins, nucleic acids etc, represented by the *Im* isotopic contrast subtraction (Table 1). We do not yet fully understand this result, that seems to imply that H_2_O dynamics in the cytoplasm are *slower* than those obtained by considering the entirety of the bacterial cell. Our *D*_*T*_ values for the various bacterial *vs* buffer solution samples are summarized in Table 2 and compared with a compilation of literature data for diffusion in H_2_O and aqueous solutions in Fig. 3.

**Figure 4:**
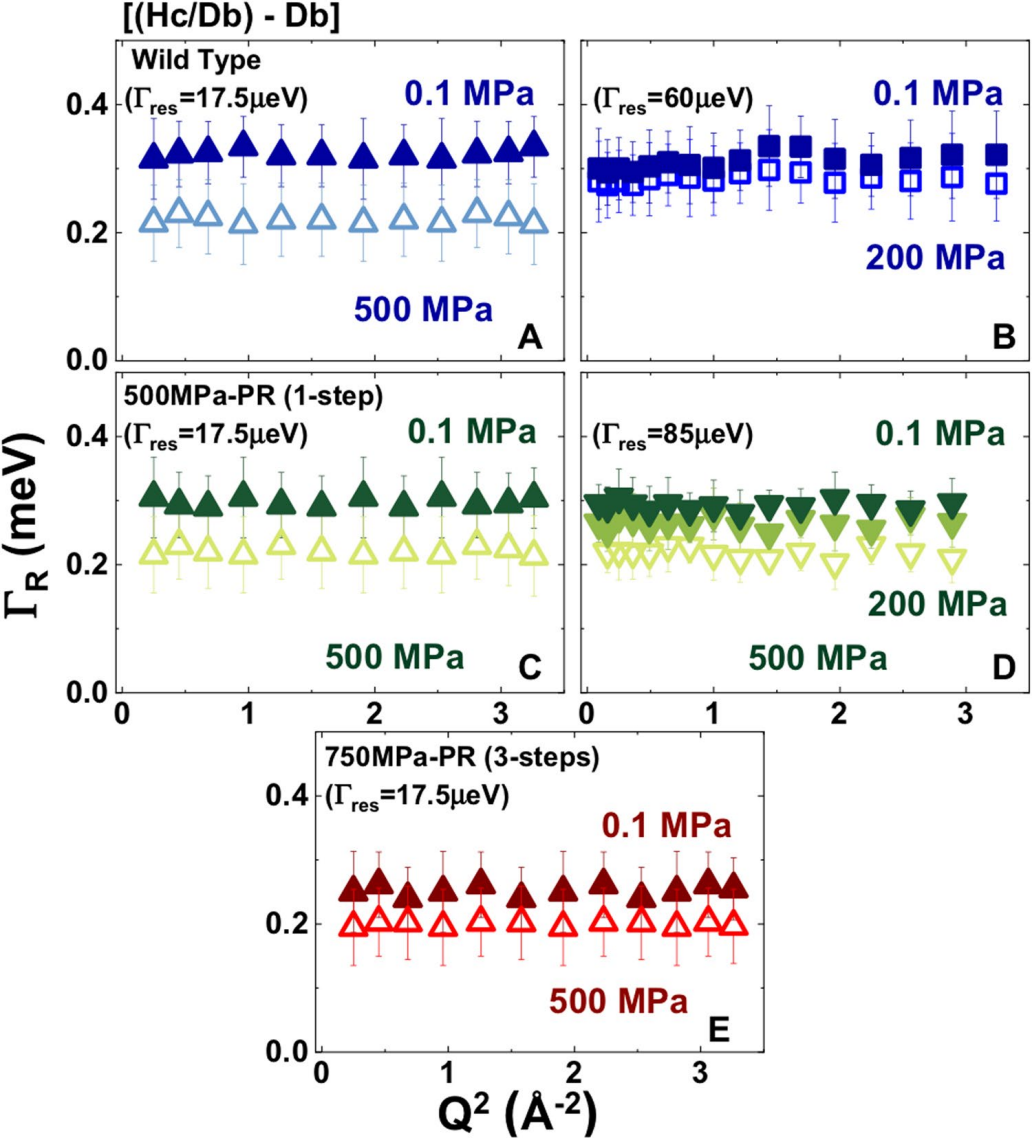
Rotational relaxation (*Γ*_*R*_*(Q*^2^*)*) data extracted from fitting QENS data for the broad Lorentzian contribution from QENS data for the dynamics of the intracellular medium (*Im* = [*Hc/Db* − *Db*]) for wild type *S. oneidensis* (top panel), 500 MPa pressure resistant (PR) survivors (middle panel) and 750 MPa PR survivors (bottom panel) recorded at room temperature at 0.1, 200 and 500 MPa. Upward pointing triangles: data obtained at IRIS (energy resolution 17.5 μeV). Square symbols: data from TOFTOF (resolution 60 μeV). Downward pointing triangles: data from IN6 (resolution 85 μeV).

**Figure 5:**
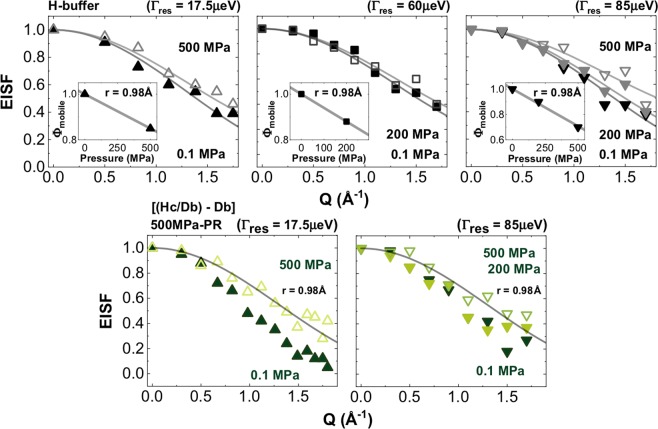
Top panels: EISF for *Hb* buffer solution, calculated using Eq. S7 (SI). The data were modelled considering roto-translational movement of H_2_O molecules with radius 0.98 Å. The inserts indicate the fraction of mobile protons detected in each experiment, on a ps timescale within the instrumental resolution window. Upward pointing triangles: data obtained at IRIS (ISIS, UK) (resolution 17.5 μeV); square symbols: data from TOFTOF (FRMII, Germany) at instrumental resolution 60 μeV. Downward triangles: data from IN6 (ILL, France) at instrumental resolution 85 μeV (λ = 5.12 Å). Bottom panels: EISF for the intracellular medium [*Hc/Db* − *Db*] for *S. oneidensis 500* *MPa PR*. The solid black line represents the model fit for *Hb* at 0.1 MPa: i.e., roto-translational movement of a molecule with radius 0.98 Å. Upward pointing triangles: data: data from IRIS (ISIS, UK): downward pointing triangles from IN6 (ILL, France).

Rotational relaxation times (*τ*_*R*_ = *ħ*/*Γ*_*R*_*; Γ*_*R*_ = 2 *ħD*_*R*_)^5,31,38,39^ extracted from the broad Lorentzian contributions are also reported in Table 2 and in Fig. 4. In addition, we analysed EISF data for our samples using a roto-translational model with the O-H distance fixed at that for water (0.98 Å) (Fig. 5). Those results revealed up to ~30% reduction in the concentration of mobile protons occurring on the ps timescale by 500 MPa.

## Discussion

The intracellular water diffusion rates determined for WT bacteria at ambient pressure obtained at the three neutron scattering facilities overlapped closely with *D*_*T*_ = 2.43–2.57 × 10^−5^ cm^2^s^−1^ (Fig. 2, Table 1). The consistency between these results provides confidence in our analysis and interpretation of the QENS datasets. The *D*_*T*_ values obtained coincide with the lower limit of measurements recorded for bulk aqueous phases, and are similar to data obtained for nanoconfined water in porous inorganic matrices^40–46^ (Fig. 3). We do note that one early QENS result reported a slowdown in water dynamics for the extreme halophile *Haloarcula marismortua* with high intracellular Cl^−^ content^47^. However, that result was not supported by more recent ^2^H NMR spin relaxation rate studies^30^.

As the pressure was increased to 200 and then 500 MPa in our experiments, the intracellular *D*_*T*_ values became increasingly lowered relative to those for bulk aqueous solutions, including the *Hb* buffer medium used to resuspend microbial samples in our experiments, decreasing to 2.00–2.11 × 10^−5^ cm^2^s^−1^ at 200 MPa and 1.76 × 10^−5^ cm^2^s^−1^ at 500 MPa (Fig. 3). The extended pressure range of our new QENS data allows to demonstrate that the intracellular water self-dynamics do exhibit significantly greater slowdown compared with bulk water and aqueous electrolyte solutions comparable to cytoplasmic compositions, with implications for bacterial functioning under highly compressive conditions relevant to deep planetary biology (Fig. 3; Tables 1, 2). We suggest that this enhanced *D*_*T*_ slowdown can be linked to the presence of spatially restricted pathways for intracellular water diffusion, leading to barriers to H_2_O mobility as the cells are compressed.

Classic interpretations of prokaryotes typically present the intracellular cytoplasm as a homogenous gel-like medium containing dissolved or suspended macromolecular and ionic components. However, recent models rather suggest a more structured internal environment, with cooperatively organized “superclustered” arrangements of proteins and macromolecular complexes, separated by channels enabling transport of the aqueous electrolyte solution^8,26–29^. Arguments for and against each view of the bacterial intracellular organisation at ambient conditions have been presented by Shepherd, who concluded that it was most useful to consider “the cytomatrix as a cooperative system of macromolecular and water networks“^8^. The significant slowdown in water mobility observed as a function of pressure, combined with the corresponding increase in rotational relaxation lifetimes, could then be associated with closing down bottlenecks, combined with increased interactions with macromolecules lining the walls of nanoscale intracellular diffusional channels^26–29^, rather than interpreted more generally as non-specific “crowding effects” within a gel-like model for the bacterial cytoplasm. Data on bulk water and dilute aqueous electrolyte solutions up to very high density show that water rotational dynamics are indeed almost unaffected by steric effects, and rather depend only on the strength and number of hydrogen bonds of the water molecule with its first neighbors^32^. We suggest that our QENS results, obtained at high pressure, are thus more consistent with channel closure within the emerging interpretation of a dynamically structured “supercluster” arrangement of macromolecular components within the cytoplasm, leaving channels for water diffusion to transport ions and hydrophilic molecules within the cell and enable reactions to occur. Our data will provide a reference point for future modelling studies.

Analysis of our EISF data for the intracellular medium (Fig. 5; Supplementary Fig. 8) compared with model fits for *Hb* at 0.1 MPa show the existence of an “extra structural contribution” to the static *vs* dynamic scattering signal. This is not surprising considering that a typical bacterial cell contains ~30% of structural macromolecules containing strongly bound H atoms (e.g., lipids, DNA, RNA, proteins etc.), that renders a fully quantitative analysis of the EISF data unfeasible. However, a qualitative comparison demonstrates an overall lowering of intensity in the high-*Q* range that points to a greater reduction in the concentration of mobile protons compared with the pure *Hb* buffer solution (i.e., ~40% *vs*. ~30%, respectively). To further investigate this effect we also examined our data for the isotopic contrast (*Dc/Hb* − *Dc/Db*) − *Hb*, that distinguishes between cytoplasmic *vs* cell envelope water dynamics^25^. This analysis was possible only for data obtained at IRIS (ISIS)^48,49^ as biomolecular dynamics recorded in the nanosecond range fall inside the energy resolution of the lower resolution spectrometers (IN6 and TOFTOF). We obtained the same result as before, with a higher proportion of mobile protons (~40%) present in the cytoplasm compared with the *Hb* buffer solution at high pressure (Supplementary Fig. 9). This observation is correlated with the greater slowdown in self-diffusion coefficient (~30 *vs*. ~20%) observed for the bacterial cells compared with bulk aqueous solutions, occurring as a result of densification.

The PR survivor populations that had been previously exposed to 500 and 750 MPa exhibited *D*_*T*_ values between 2.04–2.24 × 10^−5^ cm^2^s^−1^ at ambient pressure, that were even lower than those recorded for the WT samples. These PR samples also exhibited a greater diffusional slowdown than the buffer media as the pressure was increased to 200 and 500 MPa (Fig. 3, Table 1). The changes in *D*_*T*_ were accompanied by an average increase of ~27% in the H_2_O rotational relaxation time (*τ*_*R*_) over the same pressure ranges (Tables 1, 2; Fig. 4; Supplementary Fig. 7). This is slightly greater than *Δτ*_*R*_ ~24% observed for the *Hb* (and *Db*) buffer media, as the pressure was raised from 0.1 to 200 and then 500 MPa. Both results contrast with the relatively P-independent rotational correlation times observed for pure bulk H_2_O over the same pressure range^31^. Previous QENS studies of live bacteria including *E. coli* have found that intracellular diffusion rates are close to those of bulk aqueous solutions at ambient pressure^31^. However, our new data show that the intracellular *D*_*T*_ values occur at the lowest end of the range of previous measurements for bulk water, although they do overlap with water nanoconfined in porous environments^40–46,50^. In addition, we find that the rotational relaxation times for intracellular H_2_O differs significantly from those observed for bulk H_2_O as a function of pressure (Fig. 4, Table 1).

The pressure-induced lowering in the self-diffusion coefficient for H_2_O molecules is correlated with an increase in viscosity (*η*) and it has implications for diffusion of ions, metabolite species, biological macromolecules as well as larger nanoparticles within the cytoplasm. We can apply the Stokes-Einstein relation that links *D*_*T*_ and *η* for Newtonian fluids to estimate changes in intracellular cytosolic viscosity at the pressures and temperatures considered in this study^51,52^. At ambient P and T conditions, pure H_2_O exhibits a viscosity of 8.9 × 10^−4^ Pa  s. Our results indicate that this value should increase by 20–40% as the cells are exposed to pressures of 200 and 500 MPa. The diffusion of ions through aqueous media is expected to take place on a similar timescale to *D*_*T*_ for molecular H_2_O, whereas metabolites and proteins diffuse approximately 10–100 times slower. The relative rates of diffusion and the details of intracellular dynamics among these different species determine metabolic function as well as other important biological processes. Parry *et al*. have investigated the transport of macromolecules and other nanoscale objects through the bacterial cytoplasm and observed a size-dependent ergodicity breaking analogous to that found at the glass transition in glass-forming liquids^53^. They found that the dynamic transition between diffusive displacements and cage-like motions occurred as the particle size exceeded approximately 30 nm. The large displacement excursions of the probe molecules correlated with the metabolic activity. Although the authors discussed the cytosol in terms of a homogeneous gel-like transport medium, the arguments can be equally well applied to aqueous channels within a more highly organized model of the cellular interior structure. In that case, we might expect the “blocking” transition to be strongly pressure dependent as the nanoscale channels become constricted, and carrying out the particle tracking experiments using optical probes under high pressure conditions could be used to reveal the channel dimensions. We note that if the channels and their constrictions exhibit a range of sizes, this would have an internal filtering effect on the transport dynamics of large macromolecules and potentially foreign bodies within the bacterial cells.

We recognise that our results concern the effect of pressure on the intracellular water dynamics at ambient temperature, whereas organisms exposed to deep subsurface conditions experience a range of variable T environments. Early studies of bacterial metabolism under extreme high P were conducted at ambient T^54–56^. However, our work on survival of *E. coli* and *S. oneidensis* considered both P and T effects^36,37^. Despite the fact that *S. oneidensis* grows optimally at 30 °C at ambient pressure^57^, within the *Shewanella* genus, several strains grow and thrive in high pressure environments and at cold temperatures (e.g. *S. benthica* −50MPa and 10 °C^58^ whereas *S. frigidimarina* at ambient P and 10 °C^59^). We could not reproduce such a wide range of P and T conditions in our neutron studies. However, in our previous study, we examined the survival and colony-forming characteristics of WT and P-resistant populations at temperatures between 8 and 37 °C^36^. We did not observe any clear cross-correlation between T- and P-response effects affecting survival in *S. oneidensis*. In our neutron scattering studies, we examined the intracellular water dynamics for both wild-type (WT) and pressure-tolerant strains of *S. oneidensis* cultured following exposure to pressures extending up to 750 MPa. Specifically accounting for both P and T effects on intracellular water dynamics in different organisms is not yet possible. The temperatures in the deepest oceans and oceanic trenches where organisms have been found to survive at pressures up to 110 MPa range between −1 to + 4 °C^60^. Bulk H_2_O exhibits a density maximum and rapidly increasing viscosity throughout this range^61^, that may be reflected in the bacterial cytoplasmic dynamics, and that may influence the intracellular transport and reaction mechanisms and kinetics. This should be explored in future work combining specific targets for experimental studies of metabolic pathways linked to aqueous transport inside the cells, directed at specific model organisms. The situation in rock-hosted suboceanic or continental environments is different. Away from tectonic plate boundaries or other main thermal sources, the geothermal gradient is ~25/30 °C/Km. Once pressures of 200–500 MPa are achieved, measurements on bulk water indicate that *D*_*T*_ is almost halved (i.e., to ~1.5/1.2 10^−5^ cm^2^s^−1^, respectively)^61^. However, experiments under nanoscale confinement conditions have shown that liquid water adopts a different and thermodynamically more stable structure in the vicinity of the walls, and its dynamics change dramatically such that freezing is no longer observed: i.e., liquid water enters a “super-cold” state^40–42,44,62^. Due to the complexity of the bacterial internal structure and composition, we can not yet extend these arguments to the biophysical behavior of living organisms, but they do lead to cause for reflection.

## Conclusions

Our results demonstrate that average H_2_O diffusion rates are lower and rotational relaxation times are longer for intracellular water within the prokaryotic organism *Shewanella oneidensis* than for bulk aqueous solutions at ambient pressure. The dynamic slowdown is reflected in a decrease in the intracellular water diffusion coefficient that becomes increasingly enhanced as pressure is increased. These effects can be associated with increased constriction within nanoscale channels and interactions with the channel walls, in accord with currently emerging models of a prokaryotic cell structure with a high degree of internal organization. Our pressure-resistant bacterial survivor populations seem to exhibit slightly slower intracellular water diffusion rates than the WT samples, that could indicate more restricted pathways for H_2_O diffusion than within the general WT population. The H_2_O diffusion results are expected to correlate with increased internal viscosity of the cytosolic medium, as well as a reduction in diffusion of ions and metabolites through it. Larger macromolecules and foreign particles within the cells might become selectively blocked as the transport channels become restricted at high pressure. Our findings correlate with the observed reduction in the rates of internal metabolic processes as the pressure is increased^54–56^.

## Methods

*S. oneidensis* samples (CIP 106686) obtained from the Collection Institut Pasteur (Paris, France) were grown at 30 °C in lysogeny broth (LB) at 180 rpm, after selecting a single colony from an LB agar plate harvested at stationary phase (10^8^cells/mL). These constituted the wild type (WT) samples investigated in this study. Pressure resistant (PR) sample populations were cultured from survivors following sequential pressurization experiments in Teflon sample containers using a piston cylinder apparatus^36,37^. The *PR* samples selected for these QENS studies included one that had been treated in a single step at 500 MPa, and another that had been subjected to sequential pressurization-recovery steps at 250, 500 and then 750 MPa, after cultivation of the survivors from each run^36^. Our neutron scattering experiments examined cells with normal isotopic composition (*Hc*) as well as perdeuterated bacteria (*Dc*) that were re-suspended in normal (*Hb*) or perdeuterated (*Db*) buffer media at a concentration of 50 mg mL^−1^. We used phosphate buffer solution (PBS) at 10 mM concentration and pH7.4, containing 2.7 mM KCl and 137 mM NaCl. Perdeuterated cells (*Dc*) were prepared in the Deuteration Facility within the Life Sciences Group at the Institut Laue Langevin (ILL)^63^. The D-silantes medium used to grow the bacteria led to >98% labeling^64^. Samples were exposed to the neutron beams for 6–10 h at each pressure point. Following each experiment, recovered samples were cultured on agar plates in order to establish the survival rate through colony formation by plate counting. For the WT and PR samples, the survival rate following exposure at ambient pressure was ~80% compared with the initial concentration (Supplementary Fig. 10A). For WT bacteria exposed to 500 MPa in the beam, the survival rate was 0.05%, while that for PR samples that had been previously exposed to 500 MPa in a single step was 2.5%. The PR material that had been cultured after exposure to 750 MPa in 3 steps (0.1-250-500-750 MPa) represented 7.5% of the starting population. Survival rates following exposure to the neutron beam were comparable to those observed during *ex situ* pressurisation experiments carried out at UCL^36,37^. Complementary small angle neutron scattering (SANS) studies were carried out to ensure that extended exposure to high pressure did not result in structural modification in the bacterial samples. The similarity between the scattering profiles suggests that both *WT* and *PR* populations maintained a rod-like structure throughout these experiments (Supplementary Fig. 10B).

Room temperature QENS experiments were carried out at: IRIS (inverted time-of-flight (TOF) spectrometer)^48,49^ at ISIS (Rutherford-Appleton Laboratory, Harwell Science and Innovation campus, UK), with energy resolution 17.5 μeV; IN6 monochromatic TOF at the Institut Laue Langevin (ILL, Grenoble, France) with energy resolution 85 μeV (λ = 5.12 Å) and an extended energy range, and TOFTOF (monochromatic TOF)^65^ at the FRM-II reactor source (Garching, Germany) with an intermediate energy resolution 60 μeV (λ = 6.0 Å). The energy resolution that determined the full width at half maximum (FWHM, Γ) of the central elastic Gaussian peak was fixed by the instrument parameters and incident neutron wavelength (Supplementary Table 1). HP experiments at TOFTOF used a flat plate cell constructed from Al7075 alloy that permitted studies up to 200MPa^25,66^. The 200 and 500 MPa experiments at ISIS used a HP coil cell with internal diameter 0.5 mm, and those at ILL used a cylindrical cell; both of these were connected to a manual high pressure generator. Data were also collected at 0.1 MPa at ILL-IN6 using a flat plate Al liquid cell. Diagrams and photographs of the HP cell arrangements are given in Supplementary Fig. 11.

QENS data obtained over momentum transfers of between approximately 0.1 and 1.8 Å^−1^ provided information on diffusional and rotational water relaxation dynamics over real space correlation lengths ~3–60 Å. The large difference in incoherent neutron scattering cross section between H and D (80.27 *vs* 2.05barn, respectively) meant that D scattering was almost invisible relative to H when both were present. In our previous report of work during several experimental sessions at TOFTOF (FRM-II) we were able to examine a wide range of *Hc, Dc, Hb, Db* isotopic contrast combinations and separate out contributions from transmembrane *vs* purely intracellular (*Imc*) water dynamics. In this study we focused mainly on water dynamics within the overall intracellular environment derived from the (*Hc/Db* *−* *Db*) isotopic contrast combination (*Im*), that did not distinguish between contributions from the cytoplasm and cell envelope^25^.

Our initial experiments focused on obtaining data for *Hb* and *Db* aqueous buffer solutions, followed by QENS studies of *Hc* cells resuspended in perdeuterated buffer (*Db*). The QENS datasets were typically fit using two Lorentzian components. In agreement with previous work, we assigned the narrow component to translational diffusion with a HWHM linewidth *Γ*_*T*_*(Q*), and the broader (faster relaxing) component to a convolution of translational and rotational contributions (*Γ*_*T*_ + *Γ*_*R*_)^5,25,31,38,39,67^. The relative amplitudes of the two components were taken as free parameters to fit the lineshapes. This approach was applied to both the aqueous buffer and the *Im*, determined by the isotopic contrast (*Hc/Db* − *Db*). In cases where we had sufficient data to implement the isotopic contrast (*Dc/Hb* − *Dc/Db*) − *Hb* that focused on the cytoplasm alone (*Imc*), the datasets only supported fitting a single Lorentzian, that was mainly associated with the translational diffusion contribution.

## Supplementary information

SI_Shewanella high P H2O dynamics_revised
