# Supplementary material for: In Vivo Water Dynamics in Shewanella oneidensis Bacteria at High Pressure
Source: Sci Rep. 2019 Jun 18;9:8716. doi: 10.1038/s41598-019-44704-3 (PMC6581952; doi:10.1038/s41598-019-44704-3)
Supplement: Supplementary file 1 — SI_Shewanella high P H2O dynamics_revised [file 41598_2019_44704_MOESM1_ESM.docx]

**Supplementary information**

***In Vivo* Water Dynamics in *Shewanella oneidensis* Bacteria at High Pressure**

Fabrizia Foglia^1,*^, Rachael Hazael^2^, Filip Meersman^1,3^, Martin C. Wilding^4^, Victoria García Sakai^5^, Sarah Rogers^5^, Livia Bove^6,7^, Michael Marek Koza^8^, Martine Moulin^9^, Michael Haertlein^9^, V. Trevor Forsyth^9,10^, Paul F. McMillan^1,*^

A typical QENS signal at a given momentum exchange (*Q*) is observed as Doppler broadening in the energy transfer function as consequence of local motions and/or diffusional events. The scattering function (*S(Q,ω)*) thus contains information about the spatio-temporal correlation between identical nuclei (*S_inc_*) and the static and dynamic correlations of distinct nuclei (*S_coh_*) according to^1^:

$S\left( Q,\omega\right)=S_{inc}\left( Q,\omega\right)+ S_{coh}\left( Q,\omega\right)$ [S1]

where *S(Q,ω)* is the time Fourier transform of the intermediate scattering function (*I(Q,t)*):

$S\left( Q,\omega\right)=\frac{1}{2\pi}\int_{+\infty}^{-\infty} I\left( Q,t \right)e^{-i\omega t}dt$ [S2]

Note that because of the low *Q*-range covered in the experiment and of the high content of hydrogen (σincH = 80.27barn; σcohH = 1.76barn) in the sample, it is possible to focus on the incoherent scattering contribution. *S_inc_* can be decoupled in terms of the vibrational, translational and rotational correlations of nuclei within the sample:

$S_{inc}\left( Q,\omega\right)=S_{vib}\left( Q,\omega\right) \bigotimes S_{rot}\left( Q,\omega\right) \bigotimes S_{trans}(Q,\omega)$ [S3]

Each of these motions is assumed to be independent and is modelled by a Lorentzian function. A delta function convoluted with the instrumental resolution, determined using a vanadium standard, takes into account the frozen contributions from the sample. Note here that immobile protons (or those moving slower than the instrumental resolution), together with coherent structural components, give rise an elastic scattering (*δ(ω)*) term; while the faster dynamics are included in the flat background.

We carried out the deconvolution/subtraction procedure for each dataset and as a function of the scattering vector *Q*, separating the quasi-elastic line broadening of interest from the elastic component. Because of the large difference in incoherent scattering cross sections between H and D isotopes (σincH = 80.27barn; σincD = 2.05barn), it is also possible to highlight individual regions within the sample using contrast match technique; in these regards we assume that the quasielastic scattering from the D-component is negligible compared to the hydrogenous counterpart. Specifically we were able to separate diffusional movements associated with intracellular and extracellular water dynamics by selectively subtracting *S(Q,ω)* from each other after accounting by an appropriate amplitude scaling of these contributions (Figs. 1A-B main paper). To ensure complete H/D exchange, bacterial cells were washed (three times) and stabilised in the appropriate isotopic buffer for one hour^2^.

**Supplementary Fig. 1**. Analysis of the neutron dynamic scattering function *S(Q,ω)* for the dynamics of the intracellular medium [*Hc/Db – Db*] for *Shewanella oneidensis* wild type recorded at room temperature between 0.1 and 500MPa. Left panels: data obtained at TOFTOF (FRMII, Germany) using an instrumental resolution of 60μeV; right panels I-P: data obtained at IRIS (ISIS, UK) spectrometer with an instrumental resolution of 17.5μeV. Raw data and lineshape fits are shown with a logarithmic intensity scale to highlight QENS contributions. The central line (grey) due to elastic scattering is given by a delta function convoluted with the instrumental resolution function. For aqueous solutions both translational (blue) and translational-rotational (olive-green) Lorentzian components were used. The global fit (red continuous curve) is overlain on the data points (black).

**Supplementary Fig. 2**. Analysis of the neutron dynamic scattering function *S(Q,ω)* for the dynamics of the intracellular medium [*Hc/Db – Db*] for *Shewanella oneidensis* cultured following exposure to 500MPa (*500MPa-PR*) recorded at room temperature between 0.1 and 500MPa. Panels A-L: data obtained at IN6 spectrometer (ILL, France) spectrometer with an instrumental resolution of 85μeV; panels M-T: data obtained at IRIS (ISIS, UK) spectrometer with an instrumental resolution of 17.5μeV. Raw data and lineshape fits are shown with a logarithmic intensity scale to highlight QENS contributions. The central line (grey) due to elastic scattering is given by a delta function convoluted with the instrumental resolution function. For aqueous solutions both translational (blue) and translational-rotational (olive-green) Lorentzian components were used. The global fit (red continuous curve) is overlain on the data points (black).

**Supplementary Fig. 3**. Analysis of the neutron dynamic scattering function *S(Q,ω)* for the dynamics of the intracellular medium [*Hc/Db – Db*] for *Shewanella oneidensis* cultured following exposure to 750MPa (7*50MPa-PR*) recorded at room temperature between 0.1 and 500MPa. Data obtained at IRIS (ISIS, UK) spectrometer with an instrumental resolution of 17.5μeV. Raw data and lineshape fits are shown with a logarithmic intensity scale to highlight QENS contributions. The central line (grey) due to elastic scattering is given by a delta function convoluted with the instrumental resolution function. For aqueous solutions both translational (blue) and translational-rotational (olive-green) Lorentzian components were used. The global fit (red continuous curve) is overlain on the data points (black).

**Supplementary Fig. 4**. Analysis of the neutron dynamic scattering function *S(Q,ω)* for the dynamics of the cytoplasm medium [*(Dc/Hb – Dc/Db) – Hb*] for *Shewanella oneidensis* recorded at room temperature between 0.1 and 500MPa. Panels A-H: data relative to *Shewanella oneidensis* wild type (*WT*) obtained at TOFTOF (FRMII, Germany) using an instrumental resolution of 60μeV; panels I-T: data relative to *Shewanella oneidensis* cultured following exposure to 500MPa (*500MPa-PR*) obtained at IN6 spectrometer (ILL, France) spectrometer with an instrumental resolution of 85μeV. Raw data and lineshape fits are shown with a logarithmic intensity scale to highlight QENS contributions. The central line (grey) due to elastic scattering is given by a delta function convoluted with the instrumental resolution function. For this specific isotopic contrast only translational (blue) Lorentzian component was used. The global fit (red continuous curve) is overlain on the data points (black).

**Supplementary Fig. 5**. Analysis of the neutron dynamic scattering function *S(Q,ω)* for the dynamics of the H-buffer recorded at room temperature between 0.1 and 500MPa. Panels A-L: data obtained at IN6 spectrometer (ILL, France) spectrometer with an instrumental resolution of 85μeV; panels M-T: data obtained at TOFTOF (FRMII, Germany) using an instrumental resolution of 60μeV; panels U-AB: data obtained at IRIS (ISIS, UK) spectrometer with an instrumental resolution of 17.5μeV. Raw data and lineshape fits are shown with a logarithmic intensity scale to highlight QENS contributions. The central line (grey) due to elastic scattering is given by a delta function convoluted with the instrumental resolution function. For aqueous solutions both translational (blue) and translational-rotational (olive-green) Lorentzian components were used. The global fit (red continuous curve) is overlain on the data points (black).

**Supplementary Fig. 6**. Analysis of the neutron dynamic scattering function *S(Q,ω)* for the dynamics of the D-buffer recorded at room temperature between 0.1 and 500MPa. Panels A-L: data obtained at IN6 spectrometer (ILL, France) spectrometer with an instrumental resolution of 85μeV; panels M-T: data obtained at TOFTOF (FRMII, Germany) using an instrumental resolution of 60μeV; panels U-AB: data obtained at IRIS (ISIS, UK) spectrometer with an instrumental resolution of 17.5μeV. Raw data and lineshape fits are shown with a logarithmic intensity scale to highlight QENS contributions. The central line (grey) due to elastic scattering is given by a delta function convoluted with the instrumental resolution function. For aqueous solutions both translational (blue) and translational-rotational (olive-green) Lorentzian components were used. The global fit (red continuous curve) is overlain on the data points (black).

Our QENS profiles were analyzed using the model for water dynamics previously suggested by Sears^1,3-4^, which refers to the motion of hydrogen atoms around the molecular center of mass (i.e. oxygen atom), where the narrow Lorentzian component is purely associated to a translational diffusion, while the broader component is a convolution of translational and rotational contributions^1,3-7^.

Self-diffusion coefficients *D_T_* were evaluated by plotting the HWHM (*Γ_T_*) of the narrow Lorentzian component *vs* *Q^2^* and fitting the data using a jump model between sites separated by an average distance (*l*) with a mean residence time (*τ_0_*) during which the molecules undergo oscillatory motions:

$\Gamma_{T}=\frac{D_{T}Q^{2}}{D_{T}Q^{2}\tau_{0}+1}$ [S4]

Information about the rotational relaxation time (*τ_R_*), together with the rotational diffusion coefficient (*D_R_*), is obtained from the linewidth of the broad Lorentzian component (*Γ_T_ + Γ_R_*; representing a convolution of translational and rotational contributions).

$\Gamma_{R}= 2\hbar D_{R}=\hbar/{\tau_{R}}$ [S5]

**Supplementary Fig. 7.** *Γ_T_(Q^2^)* data extracted from fitting QENS data of the narrow Lorentzian contribution (purely translational component) from QENS data for H- (top panel) and D- (bottom panel) aqueous buffer solutions recorded at room temperature between 0.1 and 500MPa. Upward pointing triangle: data obtained at IRIS (ISIS, UK) using an instrumental resolution of 17.5μeV. Square symbols: data from TOFTOF (FRMII, Germany) using an instrumental resolution of 60μeV. Downward pointing triangle: data from IN6 (ILL, France) using an instrumental resolution of 85μeV (λ = 5.12Å).

**Supplementary Fig. 8.** *Γ_R_(Q^2^)* data extracted from fitting QENS data for the broad Lorentzian contribution (translational-rotational component) from QENS data for H- (top panel) and D- (bottom panel) aqueous buffer solutions recorded at room temperature between 0.1 and 500 MPa. Upward pointing triangle: data obtained at IRIS (ISIS, UK) using an instrumental resolution of 17.5μeV. Square symbols: data from TOFTOF (FRMII, Germany) using an instrumental resolution of 60μeV. Downward pointing triangle: data from IN6 (ILL, France) using an instrumental resolution of 85μeV (λ = 5.12Å).

QENS experiments investigate translational and/or rotational movements by mainly accounting for the incoherent scattering function, *S_inc_(Q,ω)*. This quantity contains not only information about the diffusive motion of protons (i.e., the quasielastic component) but also their position in space (elastic component). *S_inc_(Q,ω)* can then be rewritten as:

$S_{inc}\left( Q,\omega\right)=A_{0}\left( Q,T \right)\delta\left( \omega\right)+ \left( 1-A_{0}\left( Q,T \right) \right)L\left( Q,T,\omega\right)$ [S6]

where *A_0_(Q,T)* is the EISF, *δ(ω)* is the Dirac delta function representing the elastic peak and *L(Q,T,ω)* is a spectral function. This leads to the expression

$EISF(Q)=p+\left( 1-p \right)\left\{ \frac{3\left[ \sin\left( Qa \right)-\left( Qa \right)cos(Qa) \right]}{{(Qa)}^{3}} \right\}^{2}$ [S7]

where *p* is the fraction of immobile protons in the system under investigation and *a* is the O-H distance of a water molecule (a = 0.98Å). In the case of pure H-buffer (*Hb*) this model describes the system well. We note that in this case, the reduction in the percentage of mobile protons reaches a maximum of ~30% at 500MPa (Fig 5, top panel, main paper).

A different scenario emerges in the case of the analysis of intracellular media (*Im*; Fig. 5, bottom panel, main paper, and Fig. S9). Here we did not attempt to model the reported EISF because of the complexity of the system under investigation. However, a qualitative comparison serves to reinforce our interpretation of the QENS results.

**Supplementary Fig. 9.** EISF for the intracellular medium [*Hc/Db – Db*] for *Shewanella oneidensis* *WT* (panels A-B) and *500MPa PR* (panel C). Note here that in the black line in the plot represents the model fit for H-buffer at 0.1MPa (roto-translational movement of a molecule of radius of 0.98Å; Eq. S7). Upward pointing triangle: data obtained at IRIS (ISIS, UK) using an instrumental resolution of 17.5μeV. Square symbols: data from TOFTOF (FRMII, Germany) using an instrumental resolution of 60μeV.

Comparing these data with the model fits for *Hb*, we clearly see the existence of an “extra structural contribution” with a greater reduction in the percentage of mobile protons upon increasing the pressure (~40% compared with ~30% for *Im vs* *Hb* at 500MPa, respectively). This “extra structural contribution” disappears when we model the cytoplasm alone (Supplementary Fig. 10). This result is not surprising if we consider that a typical bacterial cell contains ~30% of structural macromolecules (e.g., lipids, DNA, RNA, proteins etc.).

**Supplementary Fig. 10.** EISF for the cytoplasm medium *Imc* = [*(Hc/Db) – (Dc/Db) – Hb*] for *Shewanella oneidensis* *WT* (left panel) and *500MPa PR* (right panel). The data were modelled considering a roto-translational movement of a molecule of radius of 0.98Å (Eq. S7). In the insets are reported the fraction of mobile protons. Square symbols: data from TOFTOF (FRMII, Germany) using an instrumental resolution of 60μeV. Downward pointing triangle: data from IN6 (ILL, France) using an instrumental resolution of 85μeV (λ = 5.12Å).

To ensure that our QENS profiles were accounting for live bacterial cells, we plated and counted the survivors after each run (Supplementary Fig. 11A). We note here that complementary laboratory experiments, using similar protocols, were also carried out to ensure that no cell death and/or damage were induced by prolonged exposure to neutron irradiation. Furthermore, complementary small angle neutron scattering (SANS) experiments were also performed at the SANS2D beamline (ISIS, UK) to ensure that the exposure to high pressure did not cause any structural modification in the bacterial samples. Analysis of the scattering profiles suggests that both *WT* and *PR* populations maintain their initial rod-like structure (Supplementary Fig. 11B).

**Supplementary Fig. 11.** Panel A: Bar chart showing bacterial survival. These were established following recovery to ambient pressure relative to the initial concentrations (10^8^ cells/mL) for wild type (WT) and pressure resistant (PR) samples of *Shewanella oneidensis* following neutron scattering experiment. Note that these figures are similar to the one obtained for complementary *ex situ* experiments where each sample were only exposed to pressure for the same time. Panel B: Small angle neutron scattering (SANS) data were obtained for aqueous dispersions of perdeuterated *WT* and *PR* *Shewanella oneidensis* cells in H-buffer. Data were obtained at 298 ± 0.1 K using the SANS2D beamline at ISIS (UK). The experiments were carried out for perdeuterated cells re-suspended in H-buffer medium to enhance the scattering contrast. The scattering profiles for the *WT* samples are shown in blue; *PR* samples cultured following exposure to 500 (single step) and 750MPa (3-steps) are shown as green and red symbols, respectively. I(Q) (cm^-1^) is scattering intensity, and the neutron momentum transfer Q is reported in Å^-1^.

**Supplementary Fig. 12.**  Experimental devices and arrangements for high pressure QENS experiments with live bacteria and buffer solutions. A) Flat plate Al pressure cell (5 mm thickness) used at TOFTOF (FRM-II). B) X-ray transmission image through the cell showing the 10 parallel channels (1.6 mm diameter) that contain the sample. The red square outlines the sample area examined by the neutron beam^8^. C) Cylindrical coil high pressure cell (≈ 56 mm high) used for experiments at IRIS (ISIS) up to 500 MPa; coil internal diameter = 0.5 mm thickness. The red square represents the sample area illuminated by the neutron beam. D) Parts of the IN6 (ILL) high pressure cell used up to 500 MPa before assembly. The cell body is at left and the cylindrical insert with a vertical notch to contain the sample is at bottom center. E) Filling the sample into the notched chamber using a syringe. F) The manual pressurization apparatus at IN6-ILL. The hydraulic press pumps fluid against a membrane immediately before the bacterial suspension in its aqueous buffer medium, while the sample is inside the neutron spectrometer.

| Spectrometer | Energy resolution | λ_incident_ | τ_min_ | τ_max_ |
| --- | --- | --- | --- | --- |
| IN6 | 85 μeV | 5.12 Å | ~2 ps | ~50 ps |
| TOFTOF | 60 μeV | 6.0 Å | ~2 ps | ~60 ps |
| IRIS | 17.5 μeV | - | ~10 ps | ~200 ps |

**Supplementary Table 1**. Instrumental conditions for the three sets of experiments. Spectrometers used were: i) IN6 (monochromatic TOF) Institut Laue Langevin reactor source (ILL, Grenoble, France); ii) TOFTOF (monochromatic TOF) FRM-II reactor source (Garching, Germany); iii) IRIS (inverted TOF) ISIS spallation source (Rutherford-Appleton Laboratory, Harwell Science and Innovation campus, UK).

**References**

1. Bée, M., Quasielastic Neutron Scattering: Principles and Applications in Solid State Chemistry, Biology and Material Science. Bristol: Adam Hilger (1988).
2. van Heijkamp L.F., et al. Spin-Echo Small Angle Neutron Scattering analysis of liposomes and bacteria. *Journal of Physics: Conference Series* **247**, 012016 (2010).
3. Sears V.F. Theory of cold neutron scattering by homonuclear diatomic liquids: I. Free rotation. *Can J Phys* **44**: p. 1279-1297 (1966).
4. Sears V.F. Theory of cold neutron scattering by homonuclear diatomic liquids: II. Hindered rotation. *Can J Phys* **44**: p. 1299-1311 (1966).
5. Bellissent-Funel, M.-C., S.H. Chen, & Zanotti J.-M. Single-particle dynamics of water molecules in confined space. *Phys Rev E* **59**: p. 4558-4569 (1995).
6. Teixeira J., Bellissent-Funel M.-C., Chen S.H., Dianoux A.J. Experimental determination of the nature of diffusive motions of water molecules at low temperatures. *Phys Rev A* **31**: p. 1913-1917 (1985).
7. Jasnin M., Moulin M., Haertlein M., Zaccai G., Tehei M. Down to atomic-scale dynamics inside living cells explored by neutron scattering. *J Roy Soc Interface* **6**: p. S611-S617 (2009).
8. Appavou M.-S., Busch S., Doster W., Gaspar A., Unruh T. The influence of 2 kbar pressure on the global and internal dynamics of human haemoglobin observed in quasielastic neutron scattering. *Eur J Biophys* **40**: p. 705-714 (2011).
